# Application of random survival forests in understanding the determinants of under-five child mortality in Uganda in the presence of covariates that satisfy the proportional and non-proportional hazards assumption

**DOI:** 10.1186/s13104-017-2775-6

**Published:** 2017-09-07

**Authors:** Justine B. Nasejje, Henry Mwambi

**Affiliations:** 1 School of Mathematics, Statistics and Computer Science, University of KwaZulu-Natal, 250 King Edward Avenue, Scottsville, Pietermaritzburg, 3201 South Africa; 2 School of Mathematics, Statistics and Computer Science, University of KwaZulu-Natal, Scottsville, Pietermaritzburg, 3209 South Africa

**Keywords:** Cox proportional hazards model, proportional hazards assumption, Survival trees, Random survival forests

## Abstract

**Background:**

Uganda just like any other Sub-Saharan African country, has a high under-five child mortality rate. To inform policy on intervention strategies, sound statistical methods are required to critically identify factors strongly associated with under-five child mortality rates. The Cox proportional hazards model has been a common choice in analysing data to understand factors strongly associated with high child mortality rates taking age as the time-to-event variable. However, due to its restrictive proportional hazards (PH) assumption, some covariates of interest which do not satisfy the assumption are often excluded in the analysis to avoid mis-specifying the model. Otherwise using covariates that clearly violate the assumption would mean invalid results.

**Methods:**

Survival trees and random survival forests are increasingly becoming popular in analysing survival data particularly in the case of large survey data and could be attractive alternatives to models with the restrictive PH assumption. In this article, we adopt random survival forests which have never been used in understanding factors affecting under-five child mortality rates in Uganda using Demographic and Health Survey data. Thus the first part of the analysis is based on the use of the classical Cox PH model and the second part of the analysis is based on the use of random survival forests in the presence of covariates that do not necessarily satisfy the PH assumption.

**Results:**

Random survival forests and the Cox proportional hazards model agree that the sex of the household head, sex of the child, number of births in the past 1 year are strongly associated to under-five child mortality in Uganda given all the three covariates satisfy the PH assumption. Random survival forests further demonstrated that covariates that were originally excluded from the earlier analysis due to violation of the PH assumption were important in explaining under-five child mortality rates. These covariates include the number of children under the age of five in a household, number of births in the past 5 years, wealth index, total number of children ever born and the child’s birth order. The results further indicated that the predictive performance for random survival forests built using covariates including those that violate the PH assumption was higher than that for random survival forests built using only covariates that satisfy the PH assumption.

**Conclusions:**

Random survival forests are appealing methods in analysing public health data to understand factors strongly associated with under-five child mortality rates especially in the presence of covariates that violate the proportional hazards assumption.

**Electronic supplementary material:**

The online version of this article (doi:10.1186/s13104-017-2775-6) contains supplementary material, which is available to authorized users.

## Background

The third sustainable development goal states that ensuring healthy lives and promoting the well-being for all at all ages is essential to sustainable development [1, 2]. Critical among these age groups are the children under the age of five. In 2015, the United Nations recorded that a total of 17,000 fewer children died each day than was the case in 1990. However, more than six million children still die before their fifth birthday each year. Most of these deaths occur in Sub-Saharan Africa. Uganda in particular recorded an under-five mortality rate of 71.28 per 1000 live births in the period of 2005–2011 [3]. This rate is approximately 3 times the third sustainable development goal target of at least as low as 25 per 1000 live births [4].

Identifying factors strongly associated with under-five child mortality rates is a topic of increased research interest for most of the countries in Sub-Saharan Africa, Uganda included. Several statistical methods have been used in studies aimed at identifying factors that are strongly associated with under-five child mortality rates [5–7]. Most studies have employed standard survival methodologies like the Cox-proportional hazards model [8–11]. However, the model has constantly been criticized for its restrictive assumption commonly referred to as the proportional hazards (PH) assumption [12–14].

Extensions for this model to deal with survival data in situations where the PH assumption is violated have been suggested such as the extended Cox model [15–17]. The extended Cox model is more flexible and most importantly relaxes the standard assumptions of the original Cox model, this however, comes at a cost of a more complicated model. For example, employing a smooth spline helps one to explicitly specify the functions for the Cox regression relationship but it requires one to specify correct degrees of freedom, number and placement of the knot points and order of the regression spline model (which could be quadratic, cubic, quartic, some combination of different orders, among others). In addition, polynomial spline models must be constrained by goodness-of-fit characteristics based on the actual data, resulting in penalty functions and other such criteria that cannot be universally applied to varying datasets [18–20]. This implies therefore that the hazard estimates of the extended Cox model are dependent on the parameter and model specification considered. Estimates of both nonlinearity and time-dependence vary depending upon the degrees of freedom and other parameters. Furthermore, models that fit the data equally well can have different shapes for the hazard function and result in different hazard estimates. Relying heavily on hazard estimates based on these models may require a more skilled user methodologically because there is no standardized method for determining which parameters are most appropriate [20]. However, it should be noted that when all the covariates being considered satisfy the PH assumption then the Cox PH model is preferred.

Survival trees and random survival forests formally implemented in R [21, 22], are simple but robust methods that have been considered to be an attractive alternative model choice for survival data. These methods are extensions of classification and regression trees (CART) and random forests [23, 24]. The methods are fully non parametric, have fewer assumptions and can easily deal with high dimensional data [25]. Random survival forests do not impose a restrictive structure on how the variables should be combined. If the relationship between the predictor variables and the response variable is complex with non linear patterns and interactions then random survival forests are capable of incorporating this automatically [26, 27]. Most often researchers who use the Cox PH model for time-to-event data go ahead and use it even when covariates in the model do not satisfy the PH assumption and make interpretations as if the PH assumption holds for each covariate in the model. Random survival forests do not rely on this assumption for their validity thus this can protect a user who is not familiar with model enhancements such as the extended Cox model to deal with covariates that do not satisfy the restrictive PH assumption.

In a study to identify factors strongly associated to under-five child mortality rates in Uganda [3], many of the covariates were excluded from the Cox PH model analysis due to their violation of the PH assumption. Random survival forests were recommended as alternative methods for the study [3]. These methods have been found appropriate to use in the presence of covariates that do not satisfy the PH assumption or in situations where the relationship between the response and the covariates may be complicated [26, 27]. In this study, we re-analyse the dataset used in the study by [3] using both the Cox PH model and random survival forests where the former is used to emphasize the difference between them. We also investigate the predictive performance of the two random survival forest models used in this study in the presence of covariates that violate the PH assumption and compared these results with the predictive performance of the models used in the presence of only those covariates that satisfied the PH assumption.

## Objective of the study

We implement random survival forests on Uganda Demographic Health Survey data for 2011 to determine factors strongly associated to under-five child mortality rates. First we compare the results from random survival forests with those of the Cox PH model in the presence of covariates that satisfy the PH assumption. We also fit random survival forests on our dataset including covariates that violate the PH assumption which were excluded in the first analysis [3]. We further discuss our findings on predictive performance for random survival forests in the presence of covariates that violate and those that do not violate the PH assumption.

The article is structured as follows: in the “Methods” section, we discuss the data and the methods used. The “Results” section presents results from the methods used. In the “Predictive performance” section, we present the results on predictive performance of the methods used. We state the general discussion and conclusions from this study in the “Discussion” and “Conclusions” section, respectively. Appendices 1 and 2 are provided as additional materials to describe the models and the methods used to evaluate the models, respectively.

## Methods

### Data

To understand factors affecting under-five child mortality rates in Uganda, the 2011 Uganda Demographic Health Survey (UDHS) data was used [3]. This dataset was collected from May 2011 through to December 2011. This was the fifth comprehensive survey conducted in Uganda as part of the worldwide Demographic and Health Surveys [28]. A representative sample of 10,086 households was selected during the 2011 UDHS. The sample was selected in two stages. A total of 404 enumeration areas (EAs) were selected from among a list of clusters sampled for the 2009/10 Uganda National Household Survey (2010 UNHS). In the second stage of sampling, households in each cluster were selected from a complete listing of households. Eligible women for the interview were aged between 15 and 49 years of age who were either usual residents or visitors present in the selected household on the night before the survey. Out of 9247 eligible women, 8674 were successively interviewed with a response rate of $94 \%\,(91 \%$ in urban and $95\%$ in rural areas). The study population for this analysis includes infants born between exactly one and 5 years preceding the 2011 UDHS.

### Exploratory data analysis

#### Covariates

In this study, 19 covariates are considered as candidates for analysis and their choice was based on related literature [29–31]. To some extent, other limitations like high level of missingness in the dataset influenced our covariate choice. The covariates include; mother’s age group (<20, 20–29, 30–39, 40+ years); type of residence (urban, rural); mother’s level of education (illiterate, primary, secondary and higher); partner’s level of education (illiterate, primary, secondary and higher); birth status (singleton birth, multiple births); sex of the child (male, female); wealth index (poorest, poorer, middle, richer, richest); children ever born (one child, two children, three children, four and more); birth order (first child, second to third child, 4th–6th child); religion (Catholic, Muslim, other Christians, others); types of toilet facility (flush toilet, pit latrine, no facility); mother’s occupation (not-working, sales and service, agriculture); current working status (working, not working); births in the past 1 year (no births, 1-birth, 2-births); births in the past 5 years (1-birth, 2-births, 3-births, 4-births); children under the age of five in the household (no child, one child, two children, three children, four children); sex of the household head (male, female); source of drinking water (piped water, borehole, well, surface/rain/pond/lake, others); mother’s age at first birth (less than 20, 20–29, 30–39 years). Note that all covariates are categorical. The categories of covariates that were not originally categorical, were created based on other similar studies in literature [31].

**Table 1:** The distribution of births and deaths by survival determinants

Characteristics	Dead N (%)	Alive N (%)	Total
Mother’s education level			
Illiterate Mothers	344 (7.7)	4149 (92.3)	4493
Mother completed primary	119 (6.4)	1749 (93.6)	1868
Secondary and higher	14 (4.2)	317 (95.8)	331
Partner’s level of education			
Illiterate Father	266 (7.7)	3180 (92.3)	3446
Father completed primary	170 (6.9)	2287 (93.1)	2457
Secondary and higher	41 (5.2)	748 (94.8)	789
Birth status			
Singleton births	431 (6.7)	6048 (93.3)	6479
Multiple births (twins)	46 (21.5)	167 (78.5)	213
Sex of the child			
Males	258 (7.8)	3067 (92.2)	3325
Females	212 (6.3)	3155 (93.7)	3367
Type of place of residence			
Urban	81 (5.8)	1308 (94.2)	1389
Rural	396 (7.5)	4907 (92.5)	5303
Wealth index			
Poorest	131 (7.5)	1623 (92.5)	1754
Poorer	112 (8.5)	1205 (91.5)	1317
Middle	86 (7.2)	1109 (92.8)	1195
Richer	72 (6.9)	969 (93.1)	1041
Richest	76 (5.5)	1309 (94.5)	1385
Children ever born			
One child	20 (3.3)	581 (96.7)	601
Two children	81 (7.1)	1065 (92.9)	1146
Three children	67 (6.6)	953 (93.4)	1020
Four and more	309 (7.9)	3616 (92.1)	3925
Birth order number			
First child	95 (7.6)	1154 (92.4)	1249
Second to third child	117 (5.6)	1974 (94.4)	2091
4th–6th child	149 (7.1)	1949 (92.9)	2098
6th+ child	116 (9.2)	1138 (90.8)	1254
Religion			
Catholics	217 (7.4)	2722 (92.6)	2939
Muslims	69 (7.5)	852 (92.5)	921
Other Christians	187 (6.8)	2571 (93.2)	2758
Others	4 (5.4)	70 (94.6)	74
Type of toilet facility			
Flush toilet	5 (4.1)	116 (95.9)	121
Pitlatrine	376 (6.9)	5031 (93.1)	5407
No-facility	96 (8.2)	1068 (91.8)	1164
Mother’s occupation			
Not-working	93 (6.9)	1260 (93.1)	1353
Sales and services	110 (6.5)	1589 (93.5)	1699
Agriculture	274 (7.5)	3366 (92.5)	3640
Births in past 5 years			
1-Birth	93 (4.5)	1982 (95.5)	2075
2-Birth	227 (6.5)	3288 (93.5)	3515
3-Births	140 (13.6)	887 (86.4)	1027
4-Births	17 (22.7)	58 (77.3)	75
Births in past 1 year			
No-births	309 (6.8)	4212 (93.2)	4521
1-Birth	163 (7.6)	1971 (92.4)	2134
2-Births	5 (13.5)	32 (86.5)	37
Children under 5 in household			
No-child	101 (34.9)	188 (65.1)	289
1-Child	178 (10.5)	1511 (89.5)	1689
2-Children	146 (4.9)	2831 (95.1)	2977
3-Children	35 (2.5)	1349 (97.5)	1384
4-Children	17 (4.8)	336 (95.2)	353
Mother’s age group			
Less than 20 years	29 (8.9)	296 (91.1)	325
20–29 years	235 (6.5)	3376 (93.5)	3611
30–39 years	164 (7.4)	2054 (92.6)	2218
40 years+	49 (7.9)	489 (90.1)	538
Birth order number			
First child	95 (7.6)	1154 (92.4)	1249
Second to third child	117 (5.6)	1974 (94.4)	2091
4th–6th child	149 (7.1)	1949 (92.9)	2098
6th+ child	116 (9.3)	1138 (90.7)	1254
Sex of household head			
Male	341 (6.7)	4771 (93.3)	5112
Female	136 (8.6)	1444 (91.4)	1580
Source of drinking water			
Piped water	76 (5.9)	1204 (94.1)	1280
Borehole	216 (7.3)	2731 (92.7)	2947
Well	93 (6.9)	1261 (93.1)	1354
Surface/rain/pond/lake/tank	70 (8.5)	756 (91.5)	826
Other	22 (7.7)	263 (92.3)	285
Age at first birth			
Less than 20 years	347 (7.5)	4291 (92.5)	4638
20–29 years	127 (6.3)	1899 (93.7)	2026
30–39 years	3 (12.0)	22 (88.0)	25

Table 1 shows the distribution of deaths for children under the age of five across all covariates considered in the study. The percentages of deaths for each of the covariate categories is stated in the second column of Table 1. For example, $7.7\%$ of children born to mothers with no education died before celebrating their fifth birthday. This is the highest percentage compared to those children born of mothers with primary education which is $6.4\%$ and secondary or higher education which is $4.2\%$. Covariates with categories that have the highest percentage of deaths include number of children in the household under the age of five, number of births in the past 5 years, number of births in the past 1 year, birth status and lastly age of the mother at first birth.

#### Dependent variable

Under-five child mortality rate is defined as the mortality rate from the age of 1 month to the age of 59 months. Thus the dependent variable used in our analysis is the time-to-event which in our case is the age of a child reported at the time of the interview (survey) for those still alive or the age of the child when he/she died. Thus children under the age of five that were still alive at the date of the interview were considered to be right censored.

### Analysis methods

The Cox proportional hazards model and random survival forests are both used in this analysis to identify factors that affect under-five child survival in Uganda. Two random survival forest implementations are used. The first forest is constructed on survival trees that are built using the log-rank split-rule. The second forest is constructed on survival trees built using the log-rank score split-rule. Note that the split-rule based on the log-rank score is desirable in the presence of tied event times. To evaluate the predictive performance for the models used, cross-validated integrated brier scores are used. The Cox PH model and the two random survival forest implementations are described in detail in Additional file 1: Appendix 1. To evaluate the predictive performance for the models used, cross-validated integrated brier scores are used and these are described in detail in Additional file 1: Appendix 2. Note that Appendices 1 and 2 are given as additional material in Addition file 1: Appendices 1 and 2.

## Results

### Proportional hazards analysis

#### Cox proportional hazards model

To use the Cox PH model, it is important to establish which covariates in the dataset satisfy the PH assumption. We used the Schoenfeld residual test [32–34] in R an open source software [35] using the command **cox.zph**. Under this test, it is assumed that regression parameters are constant over time, hence the corresponding hazard ratios are constant over time. All those regression parameters (covariate effects) that changed with time, do not satisfy the PH assumption and therefore do not qualify to be entered in the final Cox PH model. Note that as our first step, we fitted a Cox PH model on all covariates considered in the study and then obtained Schoenfeld residuals. Results from this analysis are presented in Table 2. Covariates that violated the PH assumption include: mother’s education level, total number of children ever born, type of residence, wealth index, birth order, number of births in the past 5 years, mother’s occupation and type of birth. These covariates were, therefore, not included in the final Cox PH analysis.

**Table 2:** Testing the proportional hazard assumption using scaled Schoenfeld residuals

Covariates	χ^2^ (df)	p-value
Mother’s education		
Illiterate	1	
Primary	4.83	0.03
Secondary and higher	7.52	<0.01
GLOBAL	11.25	${<0.01}$
Father’s education		
Illiterate	1	
Primary	0.51	0.48
Secondary and higher	0.86	0.35
GLOBAL	1.12	0.57
Sex of the child		
Male	1	
Female	1.99	0.16
Total number of children ever born		
1 child	1	
2 child	5.39	0.02
3 child	0.44	0.51
4+ child	0.26	0.61
GLOBAL	14.61	${<}0.01$
Type of place of residence		
Rural	1	
Urban	8.43	${<}0.01$
Wealth index		
Poorest	1	
Poorer	0.17	0.7
Middle	0.00	0.98
Richer	6.94	${<}0.01$
Richest	2.26	0.13
GLOBAL	9.29	0.05
Birth order		
1st	1	
2nd	0.28	0.59
3rd	6.69	${<}0.01$
4th+	2.64	0.10
GLOBAL	8.46	0.04
Age at first birth		
<20	1	
20–29	0.10	0.75
30+	0.41	0.52
GLOBAL	0.54	0.76
Previous birth interval (years)	1	
<2	Ref	
2	1.83	0.18
3	0.97	0.32
4+	2.53	0.11
GLOBAL	8.69	0.03
Number of births in the past 1 year		
No birth	1	
1 birth	0.7	0.40
2	1.24	0.27
GLOBAL	1.81	0.40
Number of births in the last 5 years		
1 births	1	
2 births	0.11	0.75
3 births	0.03	0.86
4+	5.00	0.03
GLOBAL	5.85	0.12
Mother’s age (years)		
<20	1	
20–29	0.16	0.69
30–39	0.63	0.43
40+	0.08	0.78
GLOBAL	5.58	0.13
Sex of household head		
Male	1	
Female	0.07	0.79
Source of drinking water		
Piped water	1	
Borehole	0.17	0.68
Well water	0.12	0.73
Surface/pond/lake/rain/etc	2.58	0.11
Others	1.82	0.18
GLOBAL	6.55	0.16
Mother’s occupation		
Not working	1	
Sales and Services	0.202	0.65
Agriculture	6.88	${<}0.01$
GLOBAL	14.41	${<}0.01$
Type of birth		
Single birth	1	
Multiple births	13	${<}0.01$
Religion		
Catholic	1	
Muslim	0.009	0.92
Other Christians	0.73	0.39
Others	1.59	0.21
GLOBAL	2.21	0.53

It is important to note that graphical methods can also be used to identify covariates that may potentially violate the PH assumption but are not statistical tests except for an initial exploratory assessment before a formal statistical test. Covariates with categories whose survival curves intersect or diverge disproportionately from each other over time are known to violate the PH assumption.

**Fig. 1:**
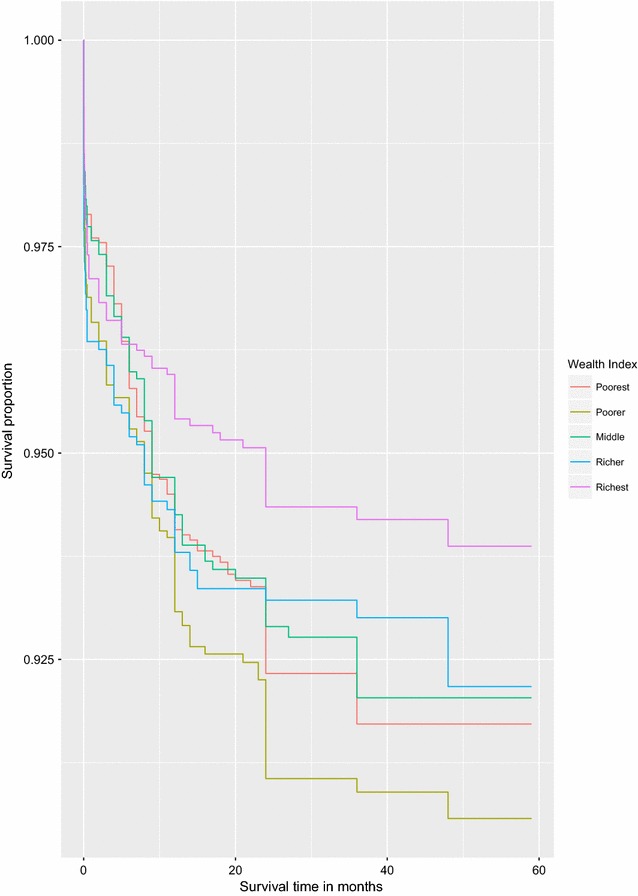
Survival curves for children under the age of five by wealth index

**Fig. 2:**
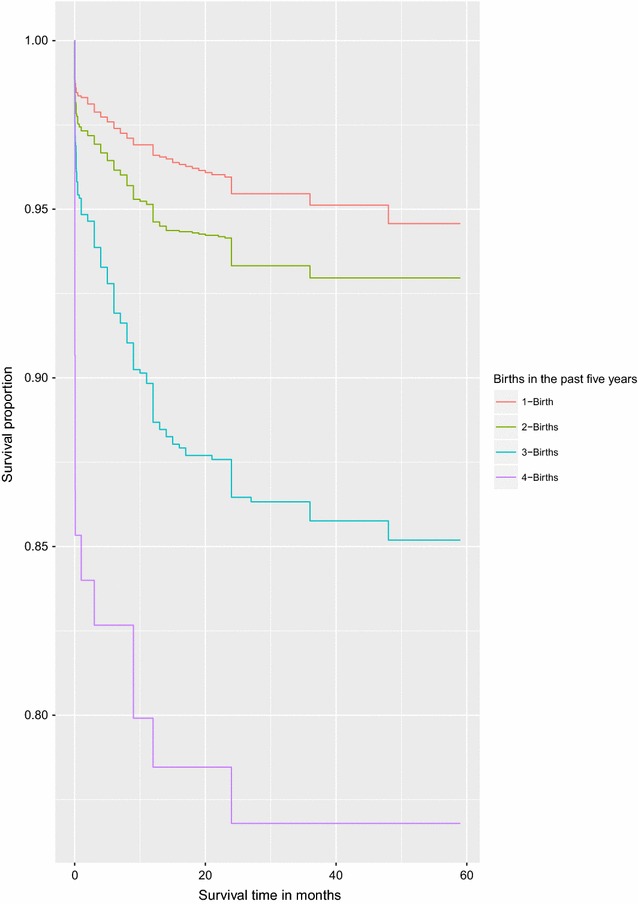
Survival curves for children under the age of five by Births in the past 5 years. Some of the survival curves diverge disproportionately from each other over time and some cross each other confirming a violation of the PH assumption (see Figs. 1 and 2)

Figures 1 and 2 illustrate a graphical method mentioned above for assessing PH assumption using two covariates that have been identified as those that violate the PH assumption. Both figures give supporting evidence to violate the PH assumption by the two covariates considered. We fitted a univariate and a multivariate Cox PH model on all covariates that did not violate the PH assumption. The results from this analysis are presented in Table 3. Sex of the child, sex of the household head and number of births in the past 1 year are the factors strongly associated with under-five child mortality rate in Uganda. The results suggest that a girl child has a $17\%$ lower hazard of death compared to the boy child. Children born in households headed by females have a $30\%$ higher hazard of death than those born in households headed by males. The results further suggest that mothers who had more than one birth in a year put their children at a higher hazard of death than those with no birth. The hazard of death for children born of mothers who had 2 births in the past 1 year was 2.34-fold higher than those born of mothers with no birth in the past 1 year. Lastly, children whose fathers had secondary and higher education were at a lower hazard of death compared to those born of illiterate fathers.

**Table 3:** The adjusted and unadjusted hazard ratios from fitting the Cox-proportional hazard model for only those covariates that satisfy the proportionality hazard assumption

Variable	Unadjusted HR [95% CI]	Adjusted HR [95% CI]	p-values
Father’s education			
Illiterate	1	1	
Primary	0.89 $\left[ 0.74, 1.08\right]$	0.92 $\left[ 0.76,1.12\right]$	0.43
Secondary and higher	0.67 $\left[ 0.48, 0.92\right]$	0.72 $\left[ 0.51 , 1.01\right]$	0.06
Sex of the child			
Male	1		
Female	0.83 $\left[ 0.69, 0.99\right]$	0.83 $\left[ 0.69,0.99\right]$	0.04
Age at first birth			
<20	1	1	
20–29	0.84 $\left[ 0.68, 1.02\right]$	0.86 $\left[ 0.69,1.06\right]$	0.16
30+	1.52 $\left[ 0.49,4.73\right]$	1.59 $\left[ 0.51,5.02\right]$	0.42
Sex of household head			
Male	1	1	
Female	1.30 $\left[ 1.07 , 1.59\right]$	1.33 $\left[ 1.09, 1.63\right]$	0.01
Number of births in the past 1 year			
No birth	1	1	
1 birth	1.18 $\left[ 0.98,1.43\right]$	1.22 $\left[ 1.01,1.48\right]$	0.04
2 births	2.34 $\left[ 0.97,5.67\right]$	2.57 $\left[ 1.06,6.25\right]$	0.04
Mother’s age (years)			
<20	1	1	
20–29	0.66 $\left[ 0.45 ,0.98\right]$	0.71 $\left[ 0.48,1.05\right]$	0.08
30–39	0.74 $\left[ 0.50,1.10\right]$	0.79 $\left[ 0.53,1.19\right]$	0.27
40+	0.90 $\left[ 0.57,1.43\right]$	0.99 $\left[ 0.62,1.59\right]$	0.98
Source of drinking water			
Piped water	1	1	
Borehole	1.24 $\left[ 0.96, 1.62\right]$	1.12 $\left[ 0.86,1.48\right]$	0.39
Well water	1.17 $\left[ 0.86 , 1.58\right]$	1.06 $\left[ 0.78,1.45\right]$	0.69
Surface/pond/lake/rain/etc	1.44 $\left[ 1.04, 1.98\right]$	1.28 $\left[ 0.91,1.79\right]$	0.15
Others	1.32 $\left[ 0.82, 2.13\right]$	1.21 $\left[ 0.75,1.94\right]$	0.44
Religion			
Catholic	1	1	
Muslim	1.01 $\left[ 0.77,1.33\right]$	1.02 $\left[ 0.77,1.34\right]$	0.91
Other Christians	0.91 $\left[ 0.75,1.11\right]$	0.94 $\left[ 0.77,1.14\right]$	0.51
Others	0.717 $\left[ 0.27,1.93\right]$	0.67 $\left[ 0.25,1.81\right]$	0.43

Using the Akaike information criteria (AIC) [36], the best fitting Cox PH model had four covariates namely: father’s education, sex of the child, mother’s age group and sex of the household head.

**Table 4:** The best fitting Cox proportional hazards model

Variable	*β* (*s.e*)	HR [95% CI]	p values
Father’s education			
Illiterate	1		
Primary	−0.09 (0.09)	0.90 $\left[ 0.75 ,1.09\right]$	0.31
Secondary and higher	−0.41 (0.17)	0.66 $\left[ 0.47,0.92\right]$	0.014
Sex of the child			
Male	1		
Female	−0.18 (0.09)	0.83 $\left[ 0.69, 0.99\right]$	0.04
Number of births in the past 1 year			
No birth	1		
1 birth	0.20 (0.09)	1.22 $\left[ 1.01,1.48\right]$	0.04
2 births	0.922( 0.45)	2.51 $\left[ 1.04, 6.09\right]$	0.04
Household head			
Male	1		
Female	0.28 (0.10)	1.33 $\left[ 1.09,1.62\right]$	0.01
Mother’s age group			
Less than 20 years	1		
20–29	−0.38 (0.19)	0.68 $\left[ 0.46 ,1.01 \right]$	0.05
30–39	−0.27 (0.20)	0.77 $\left[ 0.51,1.14 \right]$	0.17
40+	−0.05 (0.24)	0.95 $\left[ 0.59,1.51 \right]$	0.83

Results presented in Table 4 confirm that sex of the child, sex of the household head and number of births in the last 1 year were strongly associated with under-five child mortality rates in Uganda. Children whose father’s education level is secondary and higher had a lower hazard of death compared to children whose fathers were illiterate. There was no significant difference in the hazard of death for children whose fathers were illiterate or had primary education. Mother’s age group was not significant but the age groups considered gave some interesting results. Children born of mothers below 20 years of age had a higher hazard of death than those born of mothers aged between 20 and 29 years of age. There was no significant difference between the hazard of death for children under the age of five born of mothers below 20 years and those who were 40$+$ years of age. This indicates that women who give birth before 20 years of age and those who give birth after 40 years of age, put their children at an equally higher hazard of death before celebrating their fifth birthday.

We graphically illustrate the results for two of the covariates considered to be strongly associated to under-five child mortality rates in Uganda using survival curves.

**Fig. 3:**
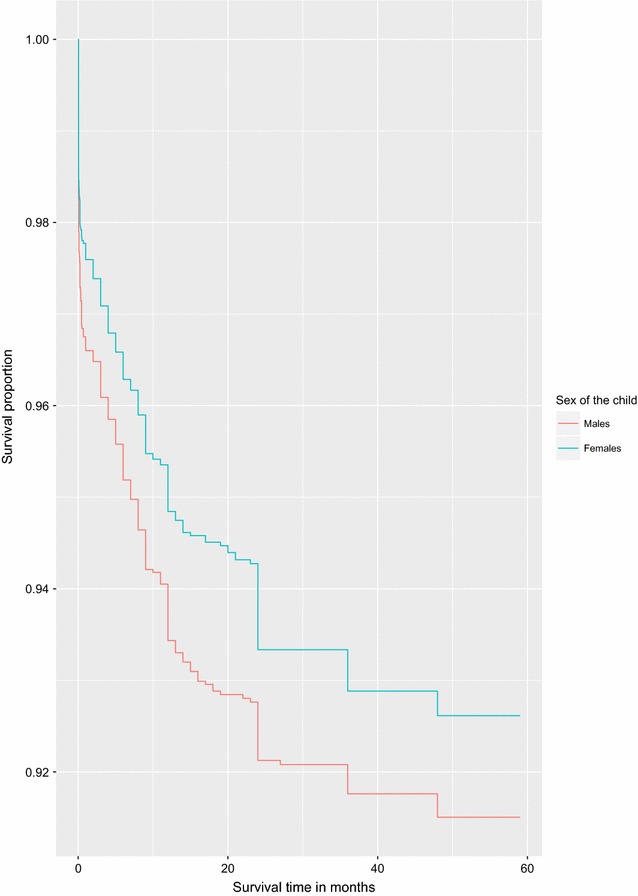
Survival curves for children under the age of five by sex of the child

**Fig. 4:**
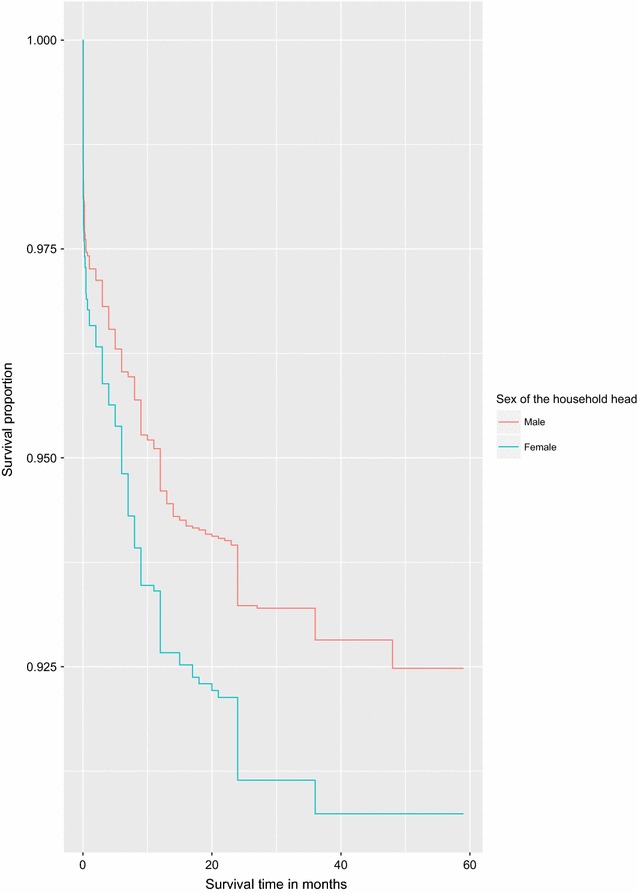
Survival curves for children under the age of five by sex of the household head

Figures 3 and 4 illustrate survival curves for the two selected covariates. The survival curve for girls is above that of boys and hence indicates a better survival rate for girls. Female headed households were also associated with a higher hazard of death for children under the age of five compared to male headed households.

#### Random survival forests built using covariates that satisfy the PH property

We fitted two random survival forest models on the dataset, that is, the one based on survival trees built using the log-rank and the log-rank score split-rules, respectively. Note that these two models were built using only covariates that were identified as satisfying the PH assumption. Characteristics of the two forests are presented in Table 5 below.

**Table 5:** Characteristics of the two fitted forests

First forest	
Number of deaths	477
Minimum terminal node size	3
Average no. of terminal nodes	514.902
No. of variables tried at each split	3
Total no. of variables	8
Splitting rule	Log-rank
Error rate	47.32
Second forest	
Number of deaths	477
Minimum terminal node size	3
Average no. of terminal nodes	607.567
No. of variables tried at each split	3
Total no. of variables	8
Splitting rule	Log-rank score
Error rate	47.36

**Fig. 5:**
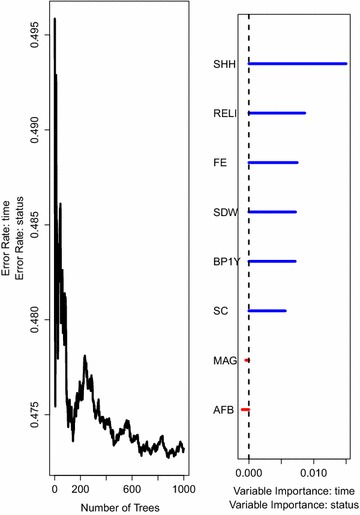
The prediction error rate (*left panel*) for random survival forest of 1000 trees together with the rank of covariates (*right panel*) based on how they influence under-five child mortality while considering covariates that satisfy the PH assumption. The trees in this forest are built using the log-rank split-rule

To identify the most important covariates in explaining survival of children under the age of five in Uganda, permutation importance was used as the measure of variable importance [22, 26, 37]. Results from fitting a random survival forest of 1000 survival trees built using the log-rank split-rule are summarised in Fig.  5. They indicate that sex of the household head (SHH), religion (RELI), father’s education (FE), source of drinking water (SDW), number of births in the past 1 year (BP1Y) and sex of the child (SC) are the most important covariates strongly associated to under-five child mortality rates in Uganda. These results are in agreement with the results obtained from fitting a multivariate Cox PH model presented in Table 3 as far as significant effects are concerned but it is interesting to note that the random survival forest model did pick other covariates as important, namely, religion and source of drinking water. The error rate for any new prediction and in this case the out-of-bag prediction error rate was $47.32\%$.

For comparison, we also fitted a random survival forest model with survival trees built using the log-rank score split-rule.

**Fig. 6:**
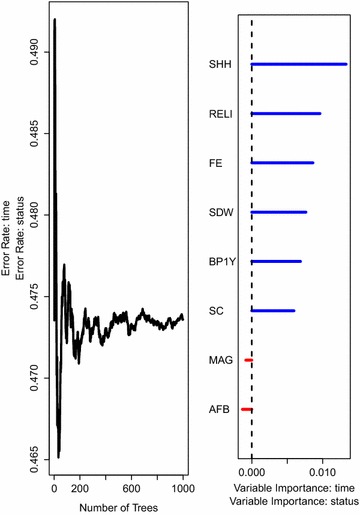
The prediction error rate* (left panel*) for random survival forest of 1000 trees together with the rank of covariates (*right panel*) based on how they influence under-five child mortality while considering covariates that satisfy the PH assumption. Survival trees in this forest are built using the log-rank score split-rule

The results on variable importance presented in Fig. 5 are similar to the results in Fig. 6. The figures further indicate that the two survival forest models have an approximately equal error rate which confirms or is in agreement with a study by [38] where the two models were found to have a similar predictive performance.

### Random survival forests built using covariates with or without the PH property

Survival trees and random survival forests divide the covariate space into subgroups of good and poor survival experience predictors. They are therefore promising methods in analysing survival data in the presence of non-proportional hazards [27]. We fitted random survival forest models under the two split rules (log-rank and log-rank score, respectively) on the 2011 Uganda Demographic Health Survey dataset. We considered all covariates in the analysis including those that violated the PH assumption. The characteristics of these two forests are presented in Table 6 below.

**Table 6:** Characteristics of the two fitted forests

First forest	
Number of deaths	477
Minimum terminal node size	3
Average no. of terminal nodes	480.167
No. of variables tried at each split	5
Total no. of variables	19
Splitting rule	Log-rank
Error rate	17.29
Second forest	
Number of deaths	477
Minimum terminal node size	3
Average no. of terminal nodes	910.187
No. of variables tried at each split	5
Total no. of variables	19
Splitting rule	Log-rank score
Error rate	19.69

The error rates from the out-of-bag sample for the forests built with survival trees based on the log-rank and the log-rank score split-rules are 17.29 and 19.69, respectively. These two error rates are much lower compared to the error rates for survival forests built based on only covariates that satisfy the PH assumption. This result confirms the improved performance of random survival forests in the presence of non-proportional hazards covariates [27]. However, making this conclusion based on the out-of-bag error rate may not be sufficient. It is also important to note that it is expected of the error rate to decrease with addition of more covariates. However, the key point in the above analysis is that the importance of covariates that satisfied and those that violated the PH assumption were evaluated.

**Fig. 7:**
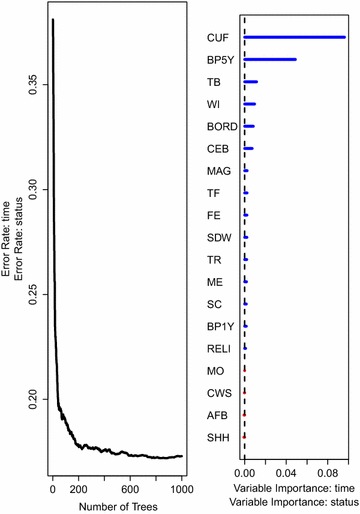
The prediction error rate (*left panel*) for random survival forest of 1000 trees together with the rank of covariates* (right panel*) based on how they influence under-five child mortality while considering all covariates including those that violate the PH assumption. Survival trees in this forest are built using the log-rank split-rule

**Fig. 8:**
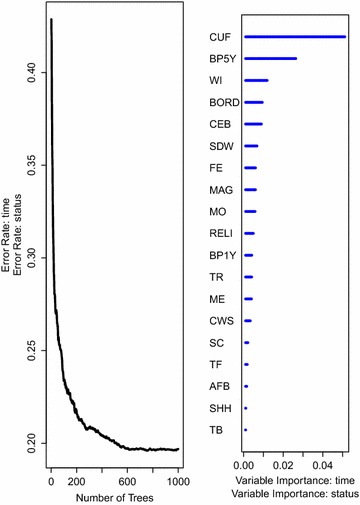
The prediction error rate curve (*left panel*) for random survival forest of 1000 trees together with the rank of covariates (*right panel*) based on how they influence under-five child mortality while considering all covariates including those that violate the PH assumption. Survival trees in this forest are built using the log-rank score split-rule

The results on factors associated with under-five mortality rate, together with the prediction error rate curves for the two random survival forest models, are presented in Figs. 7 and 8.

Results from both forests indicate that the number of children under the age of five in the household (CUF) highly influences under-five child mortality rate in Uganda. Other covariates that are strongly associated to under-five child mortality in Uganda as ranked by the forest according to their importance include: the number of births in the past 5 years (BP5Y), birth order (BORD), wealth index (WI) and the total number of children ever born (CEB). Note that the number of children under the age of five in the household had the highest percentage of death as seen in Table 1.

Covariates that were strongly associated to under-five child mortality rates in Uganda in the presence of proportional hazards show up among other covariates but do not appear to be highly ranked. This result indicates that excluding covariates in the analysis of survival data due to violation of the PH assumption leads to loss of information. We see this as a very important property for random survival forests demonstrated in these two analyses namely, the choice of covariates in the model do not need a priori to rely on the too restrictive PH assumption. This is a demonstration of flexibility on the part of random survival forests as an additional attractive property compared to models that rely on the strict PH assumption. We can, therefore, conclude that random survival forests are good alternative models to use while identifying factors affecting under-five mortality rates especially in the presence of non-proportional hazards covariates. To verify this results, we used integrated brier scores [39] as a measure of predictive performance as presented in the next section.

## Predictive performance

The predictive performance for the models used was evaluated using the integrated brier scores [39], presented in Additional file 1: Appendix 2. We used the **pec** package [40] in R [35] for this analysis. Prediction error rates of $50\%$ or higher are useless because they are no better than tossing a coin [26, 41].

**Fig. 9:**
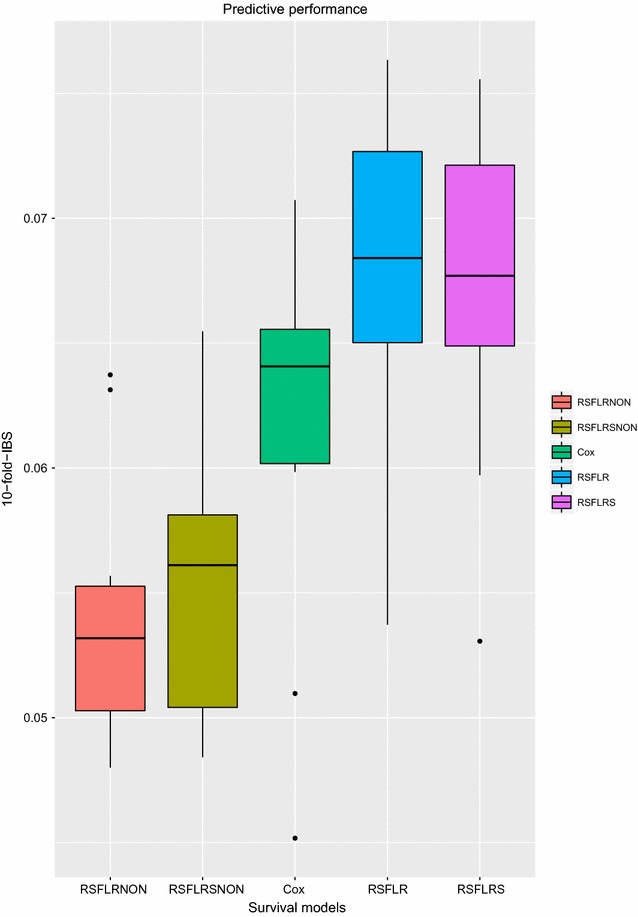
Predictive performance for random survival forests with both covariates that satisfy and violate the PH assumption, the Cox PH model and random survival forests with only covariates that satisfy the PH assumption

The results in Fig. 9 show that models used in this analysis have a good predictive performance. In the presence of non-proportional hazards covariates, random survival forest models under the two split rules (log-rank and log-rank score, respectively) show a much better predictive performance. Their predictive performance exhibited is better than that of models based strictly on the PH assumption. In the presence of proportional hazards, however, the Cox model shows a better predictive performance compared to the two random survival forests models. This strengthens the recommendation that if all covariates satisfy the PH assumption, the Cox PH model is preferable.

The good predictive performance for random survival forests in the presence of non-proportional hazards covariates is an appealing result in the analysis of survival data especially that from public health. This is because covariates with non-proportional hazards have often been excluded in the analysis of survival data especially when the standard Cox proportional hazards model was being used for analysis. In some cases, other models like the extended Cox model have been used but they are known to have some restrictive formulation complexities. Using a stratified Cox PH model is another alternative to dealing with covariates that do not satisfy the PH assumption. However, the downside of this approach is that if a covariate is used as a stratifying variable its effect on the outcome cannot be estimated yet a researcher(s) might be interested in its effect. Random survival forests are flexible and have fewer assumptions. They are, therefore, plausible alternative models in analysing survival data to understand factors affecting under-five mortality rates in the presence of proportional and non-proportional hazards. However, further research is required on the merits and demerits of the methods.

## Discussion

Survival trees and random survival forests are increasingly becoming popular alternative models for the analysis of time-to-event outcomes [42]. They have been identified as suitable models in analysing survival data in situations where the proportional hazards assumption is violated [27, 43]. However, not much literature is available to confirm the assertion. In this study, we have therefore compared the predictive performance of the Cox proportional hazards model to the random survival forests by re-analysing a dataset that was first analysed by [3]. The study further compares the performance of random survival forests on the same dataset in the presence of covariates that violate the proportional hazards assumption to that when these covariates are excluded. Under the PH assumption, the three models show that sex of the household head, sex of the child and the number of births in the past 1 year are strongly associated to under-five child mortality rate in Uganda.

Other covariates such as source of drinking water, Father’s education and religion show up as important in explaining under-five child mortality rates in Uganda with random survival forest models. However, these covariates did not appear to be very strongly associated to under-five child mortality rate in the Cox proportional model. It is interesting to note that random survival forest models give additional information in regard to variable importance.

Results from the two forest models in the presence of non-proportional hazards show that the number of children under the age of five in a household, greatly influences under-five child mortality rates. This ranks top in the two random survival forest models. Other factors ranked as important in understanding under-five child mortality rates by random survival forests in the presence of non-proportional hazards covariates are: births in the past 5 years, wealth index, birth order and total number of children ever born. Similar factors have emerged to be strongly associated to under-five child mortality rates in other studies [3, 29, 44, 45].

To compare the predictive performance of these three models on the scenarios considered, we used integrated brier scores via cross-validation. The Cox proportional hazards model had a better predictive performance in the presence of only those covariates that satisfy the proportional hazards assumption compared to the two random survival forest models. This result may not be seen as a surprise because the Cox PH model works best under this assumption from which its original formulation by [8] is based. The result is further confirmed because the two random survival models had a high out-of-bag error rate of 47.36 and 47.32%, respectively. The out-of-bag error rate for the two random survival forest models (RSFLR, RSFLRS) in the presence of proportional hazards are higher compared to those of random survival forest models (RSFLRNON, RSFLRSNON) in the presence of non-proportional hazards covariates. This implies that excluding covariates that have non-proportional hazards in the analysis gives less informative results. The results further confirm that random survival forests are robust in approximating complex survival functions, including functions based on covariates with non-proportional hazards, while maintaining low prediction error rates [27, 46–48].

However, since most aspects of these models are under development, it is recommended that one uses them hand in hand with the standard methods like the Cox proportional hazards model. The same recommendation was made in other studies related to random forests [42, 47, 49, 50]. It has also been established that random survival forests are useful in situations where the relationship between the response and the predictors may be complicated [26]. However, there are concerns that survival trees are built using the log-rank split-rule whose power to discriminate between two groups is highest when the proportionality hazards assumption holds. This may have an impact on the predictive performance of the survival forest model. This is important especially when the survival (or hazard) functions cross each other in the two groups being compared [51]. However, more research is needed to fully ascertain this fact especially in the presence of non-proportional hazards. More research will also guide scholars to the best split-rule that may help in such circumstances. A recent study [51] has recommended the use of the integrated absolute difference between the two daughter nodes’ survival functions as the splitting rule in circumstances where the hazard function cross. They have concluded that forests built with this rule produce very good results in general, and that they are often better compared to forests built with the log-rank splitting rule.

## Conclusions

The study confirms that random survival forests have a good predictive performance in the presence of non-proportional hazards [27]. It is, therefore, clear that these methods are promising alternatives to models that rely heavily on the proportional hazards assumption where the presence of covariates that violate the proportional hazards assumption is inevitable.

This study has demonstrated that the Cox PH model and random survival forests could cleverly be used in a complementary manner to fully model and analyse survival data in the presence of proportional and non-proportional hazards. The good predictive performance shown by the two random survival forest models in the presence of non-proportional hazards covariates for this dataset implies that these models could be alternative models in analysing survival datasets especially when the assumption is violated. Our conclusions on the use of random survival forests to analyse survival data are in agreement with the recommendations by [26, 50]. Obvious extensions that came to light when dealing with large survey data is when there are outcomes and covariates with missing data. We propose combining random survival forests with multiple imputation methods to reduce the loss of information. The combined approach will be to apply random survival forests after multiple imputation. A limitations to this study is that we have used random survival forest models that have been identified to favour to covariates with many split points in survival tree building [52–55]. Given the fact that most of our covariates were categorical with more than two categorises, biased results on estimates such as variable importance are inevitable [53, 55]. Our recent study[56] has therefore recommended the use of conditional inference forests suggested by [57] in the presence of covariates with many split points.

## Additional file

**Additional file 1.** Methods and model evaluation techniques. The file contains algorithms for the methods used in this study together with their evaluation technique.

## Abbreviations

PH: proportional hazards
HR: hazard ratio
CART: classification and regression trees
UDHS: Uganda Demographic Health Survey
SHH: sex of the household head
RELI: religion
FE: Father’s education
SDW: source of drinking water
BP1Y: number of births in the past one year
SC: sex of the child
CUF: number of children under the age of five in the household
BP5Y: number of births in the past five years
BORD: birth order
WI: wealth index
CEB: total number of children ever born
RSFLR: random survival forests with log-rank split-rule on data with proportional hazards
RSFLRS: random survival forests with log-rank score split-rule on data with proportional hazards
RSFLRNON: random survival forests with log-rank split-rule on data with both proportional and non-proportional hazards
RSFLRSNON: random survival forests with log-rank score split-rule on data with both proportional and non-proportional hazards
OOB: out-of-bag
IBS: integrated brier scores
