# Supplementary material for: Application of random survival forests in understanding the determinants of under-five child mortality in Uganda in the presence of covariates that satisfy the proportional and non-proportional hazards assumption
Source: BMC Res Notes. 2017 Sep 7;10:459. doi: 10.1186/s13104-017-2775-6 (PMC5590231; doi:10.1186/s13104-017-2775-6)
Supplement: Supplementary file 1 — Additional file 1. Methods and model evaluation techniques. The file contains algorithms for the methods used in this study together with their evaluation technique. [file 13104_2017_2775_MOESM1_ESM.pdf]

Methods and model evaluation techniques

Appendix 1

Method 1

Cox proportional hazards model

The Cox model [**cox1972regression**] has the form:

$$h\left(t|X\right) = h_0\left(t\right)\exp\left(X^T\beta\right) \ . \quad (1)$$

The measure of the effect of any given covariates on survival time is given by the hazard ratio denoted as HR. Using a categorical variable with two levels, say $X = 1$ and $X = 0$, the hazard ratio for the two groups is defined as:

$$HR = \frac{h\left(t|X = 1\right)}{h\left(t|X = 0\right)} = \exp\left(\beta\right) \ . \quad (2)$$

$HR = 1$, indicates that individuals in the two categories are at the same hazard of experiencing the event. $HR > 1$ indicates that individuals in category $(X = 1)$ are at a high hazard of experiencing the event. Lastly, when $HR < 1$, individuals in category $(X = 0)$ are at a high hazard of experiencing the event. To use the Cox-proportional hazard model, all the covariates entered in the model must satisfy the proportional hazards assumption. This implies that the model can give invalid results in situations where the PH assumption is violated.

Method 2

Survival trees and random survival forests

Survival trees [**gordon1985tree**] have been increasingly recommended by researchers as
alternatives to the Cox model [**ciampi1987recursive**, **segal1988regression**]. The algorithm for building survival trees for random survival forests[**ishwaran2008random**, **breiman2001random**] is given in Algorithm 1 below.

The split-rule mentioned in step 2 of the algorithm is very important in tree building. In this article, we use the log-rank and the log-rank score split-rules.

Algorithm 1 : Survival tree algorithm

1: At each node randomly select $\sqrt{p}$-covariates from p-covariates as candidates for splitting the node into two daughter nodes.
2: Compute the impurity measure based on a predetermined split-rule at the node on each covariate selected in step 1.
3: Split the node into two daughter nodes (α and β) using the value of the impurity measure. The best covariate split maximizes the difference between the two daughter nodes.
4: Recursively repeat steps 2 and 3 by treating each daughter node as a root node.
5: Stop if a node is terminal, i.e., has no less than $d_0 > 0$ unique observed events.

The log-rank split-rule

Suppose a node h can be split into two daughter nodes α and β. The best split at a node h, on a covariate x at a split point c^* is the one that gives the largest log-rank statistic between the two daughter nodes [**ciampi1987recursive**]. The log-rank statistic for a split on x at a given covariate value c^* is defined as:

$$i(x, c^*) = \frac{\sum_{j=t_1}^{t_N} \left(d_{\alpha,j} - E\left(D_{\alpha,j}\right)\right)}{\sqrt{\sum_{j=t_1}^{t_N} \text{Var}\left(D_{\alpha,j}\right)}} , \tag{3}$$

where $d_{\alpha,j}$ is the number of events in daughter node α at time point j. The expected number of events in daughter node α, $E\left(D_{\alpha,j}\right)$ and its variance are given by:

$$E\left(D_{\alpha,j}\right) = R_{\alpha,j} \frac{d_j}{R_j} ,$$
$$\text{Var}\left(D_{\alpha,j}\right) = \frac{R_{\alpha,j}}{R_j} \left(1 - \frac{R_{\alpha,j}}{R_j}\right) \left(\frac{R_j - d_i}{R_j - 1}\right) d_j ,$$

where d_j is the total number of observed events at time point j. $R_{\alpha,j}$ is the number of individuals at risk in node α at time point j and R_j the combined number at risk in daughter nodes α and β. The algorithm for building a survival tree using the split-rule based on the log-rank statistic is given in Algorithm 2 below.

Algorithm 2 : The log-rank survival tree algorithm

1: At each node randomly select $\sqrt{p}$-covariates from p-covariates as candidates for splitting the node into two daughter nodes.
2: At a node h, compute the log-rank statistic impurity measure defined above for daughter nodes α and β formed by all possible splits on all covariates considered for splitting at the node.
3: Choose the covariate that has the largest significant log-rank statistic calculated from one of the daughter nodes created by the splits. Partition the node into two daughter nodes based on the values of the covariate obtained from the split with the largest statistic.
4: Recursively repeat steps 2 and 3 by treating each daughter node as a root node.
5: The node is terminal if it has no less than $d_0 > 0$ unique observed events.

The log-rank score split-rule

The log-rank score split-rule [**hothorn2003exact**] is a modification of the log-rank split-rule defined above. It uses the log-rank scores [**lausen1992maximally**]. Given $r = (r_1, r_2, \dots, r_N)$, the rank vector of survival times with their indicator variable

$$(T, \delta) = ((T_1, \delta_1), (T_2, \delta_2), \dots, (T_N, \delta_N)) \ ,$$

and that $a = a\,(T, \delta) = (a_1(r), a_2(r), \dots, a_N(r))$ denotes the score vector depending on ranks in vector r. Assume that the ranks order the predictor variables in such a way that $x_1 < x_2 < \dots < x_N$. The log-rank scores for an observation at T_l is given by:

$$a_l = a_l\,(T, \delta) = \delta_l - \sum_{k=1}^{\gamma_l(T)} \frac{\delta_l}{N - \gamma_k(T) + 1} \ ,$$

where

$$\gamma_k(T) = \sum_{l=1}^{N} \chi\{T_l \leqslant T_k\}$$

is the number of individuals that have died or censored before or at time T_k. The log-rank score statistic is defined as:

$$i\,(x, c^\star) = \frac{\sum_{x_j \leq c^\star} (a_j - R_1 \bar{a})}{\sqrt{R_1 \left(1 - \frac{R_1}{N}\right) S_a^2}} \ , \tag{4}$$

where $\bar{a}$ and S_a^2 are the mean and sample variance of the scores $\{a_j : j = 1, 2, \dots n\}$. The best split is the one that maximizes $|i\,(x, c^\star)|$ over all $x_j^{'}$s and possible splits $c^\star$.

Random survival forests

Generally, trees are unstable and hence researchers have recommended the growing of an entire forest [**breiman2001random**, **dietterich2002ensemble**]. Random survival forests [**ishwaran2008random**, **ishwaran2014randomforestsrc**] are considered to be the solution to the problems of using a single survival tree. The random survival forests algorithm implementation [**ishwaran2008random**] is given as:

Algorithm 3 : Random survival forest algorithm

1: Draw B bootstrap samples from the original data set. Each bootstrap sample excludes about 30% of the data and this is called out-of-bag (OOB) data.
2: Grow a survival tree for each bootstrap sample. At each node randomly select $\sqrt{p}$ from p covariates as candidates for splitting. Split the node by selecting the covariate that maximizes the difference between daughter nodes using a predetermined split rule.
3: Grow the tree to full size under the constraint that a terminal node should have no less than $d_0 \ > 0$ unique deaths.
4: Calculate the cumulative hazard (CH) for each tree. Average to obtain the ensemble prediction.
5: Using OOB data, calculate prediction error for the ensemble cumulative hazard.

Appendix 2

Model evaluation techniques

Integrated Brier scores **[graf1999assessment]** are used to compare the predictive performance of all the split-rules used in random survival forests for this study. At a given time point t, the Brier score for a single subject is defined as the squared difference between observed survival status (e.g., 1=alive at time t and 0=dead at time t) and a model based prediction of surviving time t . Using the test sample of size N_{test}, Brier scores at time t are given by:

$$\begin{aligned} BS\left(t\right) = \frac{1}{N_{\text{test}}}\sum_{l=1}^{N_{\text{test}}} \Bigg\{ \left[0-\widehat{S}\left(t|x\right)\right]^2 \frac{I\left(t_l \leq t, \delta_l = 1\right)}{\widehat{G}\left(t_l|x\right)} \\ + \left[1-\widehat{S}\left(t|x\right)\right]^2 \frac{I\left(t_l > t\right)}{\widehat{G}\left(t|x\right)} \Bigg\}\,. \end{aligned} \tag{5}$$

Where $\widehat{G}\left(t|x\right) \approx P\left(C > t|X = x\right)$ is the Kaplan-Meier estimate for the conditional survival function of the censoring times.
The integrated brier score(IBS) are given as:

$$IBS = \int_0^{\max(t)} BS\left(t\right) dt\,.$$

It is common practice to hold out part of the available data to validate the model. This is done to avoid the problem of overfitting that arises from using the same dataset to train and test the model. The available data however, is often not large enough to divide it into the test and train data set. We therefore used a $10-$fold cross-validation approach where the data set is split into 10 datasets of approximately equal size and the IBS is calculated on each fold left-out while training the model on the other 9 datasets.
